# Bridging policy gaps in clinical trial volunteering: public and healthcare professional perspectives in England

**DOI:** 10.1136/bmjophth-2026-002903

**Published:** 2026-07-28

**Authors:** Khawaja Muhammad Ammar Ali Javed, Gibran F Butt, Gurpreet Bansal, Maryam Noeman, Philip I Murray, Saaeha Rauz

**Affiliations:** 1 Academic Unit of Ophthalmology, University of Birmingham, Birmingham, UK; 2 Ophthalmology, Birmingham and Midland Eye Centre, Birmingham, UK

**Keywords:** Public health, Inflammation, Experimental & laboratory, Clinical Trial

## Abstract

**Objective:**

To examine organisational leave policies for clinical trial participation in England and compare willingness between the public and healthcare professionals (HPs) under varying leave conditions.

**Methods and analysis:**

Freedom of information (FOI) requests were submitted to 378 public-sector organisations to identify policies supporting leave for clinical trial participation. Parallel cross-sectional surveys of the public (n=503; working-age adults) and HPs (n=110) assessed willingness to participate as a healthy volunteer (HV) or patient (P) in scenarios requiring annual leave (AL) or unpaid leave (UL). Free-text responses were analysed thematically. Group comparisons used χ² tests, and multivariable logistic regression examined predictors of willingness, adjusting for demographic and employment characteristics.

**Results:**

Five organisations (1.3%) reported an explicit policy addressing clinical trial leave while 336 (88.9%) reported none. Across all scenarios, public respondents were more willing to participate than HPs, particularly for HV participation using AL (78.9% vs 63.6%; p=0.001). In adjusted analyses, HPs had lower odds of willingness than public respondents for HV–AL (aOR 0.52, 95% CI 0.32 to 0.85), P–AL (aOR 0.67, 95% CI 0.44 to 0.98) and P–UL (aOR 0.61, 95% CI 0.38 to 0.96). Willingness declined when UL was required. No association was observed between Index of Multiple Deprivation decile and willingness. Financial concerns were the main barrier among the public, whereas HPs cited limited availability of AL.

**Conclusions:**

There is a widespread lack of organisational policies supporting clinical trial participation leave, and willingness is highly sensitive to whether leave is paid. This is particularly relevant in ophthalmology, where trials often involve repeated, time-intensive visits and reliance on working-age carers. Limitations include non-probability sampling, uncalculated survey response rates, self-reported willingness, a small regionally recruited HP sample and restriction to public-sector FOI data. Employment policy represents an under-recognised structural determinant of clinical trial participation, with implications for ophthalmology research.

**WHAT IS ALREADY KNOWN ON THIS TOPIC**

- Clinical trial recruitment is significantly influenced by participant burden, time commitment and financial considerations, particularly in studies that require repeated visits. Previous research has focused largely on patient-level and study-design barriers, with limited attention to the role of employer leave policies in shaping willingness to participate. Ophthalmology trials often involve high visit frequency and carer involvement, making employment-related constraints particularly relevant.

**WHAT THIS STUDY ADDS**

- This study demonstrates a near-complete absence of explicit clinical trial leave policies across English public-sector organisations and shows that willingness to participate is strongly influenced by whether leave is paid or unpaid. It provides the first comparative assessment of public and healthcare professional perspectives on trial participation under different leave conditions, grounded in real-world employment contexts.

**HOW THIS STUDY MIGHT AFFECT RESEARCH, PRACTICE OR POLICY**

- The findings highlight employment policy as an under-recognised structural determinant of clinical trial participation, with potential implications for ophthalmology research. Introducing clear, equitable provisions for research-related leave and standardised reimbursement could reduce barriers, improve recruitment and retention and may promote fairer access to participation in clinical trials.

## Introduction

 Despite extensive research on patient-level and study-level barriers, the role of employment policy in shaping participation in clinical trials remains largely neglected.^1–3^ Trial participation often requires attending study visits during working hours, yet most employees in the UK lack any statutory entitlement to leave for this purpose.^4^ The lack of such protections means that participation is often contingent on an individual’s ability to forgo income or use annual leave (AL). These challenges are noticeably apparent in ophthalmology, where clinical trials commonly require repeated hospital visits, prolonged follow-up and specialised imaging or visual-function testing that must be undertaken during standard working hours.

The urgency of addressing barriers to trial participation has grown in the post-COVID landscape, where the recovery of research infrastructure and public trust is paramount. The National Institute for Health and Care Research (NIHR) has identified recruitment shortfalls as a critical bottleneck, with many trials failing to meet enrolment targets despite increased investment and streamlined approvals.^5 6^ As the UK aims to become a global leader in clinical research, addressing overlooked structural barriers, such as employment leave policies, is essential to ensure inclusive, efficient and equitable trial participation. This study was motivated by direct experience of conducting investigator-led early-phase clinical trials within NHS research settings, where recruitment and retention challenges were frequently linked to work-related constraints affecting participants or their carers and to uncertainty regarding employer support. These observations arose in the context of registered studies (ISRCTN: 32614942; ISRCTN: 99747720). These registered studies provided the practical motivation for the present work, but no patient-level or recruitment data from these trials were analysed in this manuscript. This study centres on the employment-policy dimension of clinical-trial participation using a mixed-methods approach to explore how leave arrangements and employer policies shape willingness to participate. It triangulates evidence from three sources: (1) Freedom of Information (FOI) requests to public-sector organisations on trial-leave policies; (2) a survey of public respondents in England and (3) a parallel survey of healthcare professionals (HPs).

UK employment law provides statutory protection for jury service: employees are entitled to time off, and employers cannot dismiss or penalise them for attending court, with compensation for lost earnings and expenses.^7^ Since April 2024, employees have also been entitled to 1 week of unpaid carer’s leave annually^8^ and may take time off for dependants in emergencies. However, there is no general legal right to time off for medical appointments, except for antenatal care,^4^ unless taken as sick leave. This policy disparity contrasts with how other socially valuable activities are treated. Blood donation is widely promoted as a civic good and supported through national campaigns and workplace initiatives, but it similarly depends on employer goodwill rather than any statutory entitlement to paid leave.^9 10^ By contrast, jury service is protected as a duty of citizenship. Yet clinical trial participation, despite being fundamental to evidence-based medicine, receives no equivalent recognition or protection. This omission is notable in specialties such as ophthalmology, where trial participation often depends on sustained engagement over months or years and where employment constraints may play a decisive role in feasibility.

Recruitment into clinical trials remains one of the most persistent challenges in research. An estimated 40%–60% of trials fail to meet recruitment targets, and nearly one in five are terminated prematurely due to inadequate enrolment.^5 11^ Fewer than 60% of publicly funded UK randomised control trials (RCTs) achieve planned sample sizes,^6^ leading to underpowered results and wasted public funds.^12 13^

Barriers are multifactorial: fear of side effects, mistrust, logistical constraints and competing work/family demands.^14–16^ Complex protocols and frequent visits deter participants,^17^ and while interventions such as communication improvements and financial reimbursement help, evidence remains fragmented.^18^

The absence of formal protections has equity implications. Participation is easier for those in flexible or secure employment, whereas individuals in lower paid or precarious work face disproportionate barriers. The NIHR INCLUDE framework highlights the need to recruit underserved groups.^19^ Analyses of UK RCTs confirm that participants are disproportionately drawn from less-deprived areas.^20 21^ These inequities prompted the investigation into whether employer policies themselves may be a hidden structural barrier.

The study seeks to: (1) map employer policies supporting trial leave; (2) compare willingness across public and HP cohorts under annual versus unpaid leave (UL) scenarios; and characterise perceptions, barriers and incentives.

## Methods

### Study design and setting

This mixed-methods study combined organisational policy review with cross-sectional survey data. To identify public-sector policies supporting leave for clinical trial participation, FOI requests were submitted under the UK Freedom of Information Act 2000.^22^ Two complementary surveys of the general public and HPs were conducted to compare willingness to participate across different leave scenarios.

Both surveys used non-probability sampling, with recognised limitations in representativeness and response rate estimation. Study materials, including the FOI template, organisational responses and survey instruments, are provided in online supplemental appendices. Reporting followed the Standards for Reporting Qualitative Research (SRQR) guideline^23 24^ (online supplemental appendices 1–4).

### FOI requests

FOI requests were sent to 378 organisations: 330 local authorities in England and 48 targeted West Midlands or regional organisations, including NHS bodies, universities, academy trusts, police, fire and other public-facing organisations. Organisations were identified through publicly available directories and organisational websites to provide broad coverage of local authorities and major regional employers.

Requests were issued in November 2021, with responses required within 20 working days under the Freedom of Information Act 2000.^22^ Non-responders were followed up and given an additional 2 weeks.

### Survey development and content

Both surveys collected demographic data (age, gender, postcode, ethnicity) and working patterns. Individuals aged >65 years were excluded from the public survey to reflect the typical retirement age, whereas HPs were not age restricted.

Socioeconomic status was assessed using the English Index of Multiple Deprivation (IMD) 2019 deciles,^25^ derived from postcodes and treated as a continuous variable (1=most deprived, 10=least deprived).

Respondents reported willingness to participate as a healthy volunteer (HV) or patient (P) in two scenarios: AL or UL. Additional items assessed awareness of employer policies and perceived barriers.

### Survey distribution

The public survey was administered via Prodege, a consumer marketing and insights platform (www.prodege.com) on the Pollfish platform (https://www.pollfish.com) from 14 March 2022 to 27 March 2023. Pollfish distributes surveys through a network of mobile applications, inviting users to participate in-app while on their devices. Participants are recruited from a pre-existing panel of users who have opted in to receive survey invitations, typically in exchange for small incentives such as in-app rewards or access to content. Invitations are delivered dynamically using a randomised algorithm based on predefined eligibility criteria, and ethnic quotas are applied to enhance sample diversity. As recruitment and distribution were managed by the platform, the total number of individuals exposed to the survey and the response rate could not be determined; the resulting sample is, therefore, considered a convenience sample.

The HP survey was distributed via Research Electronic Data Capture (REDCap),^26 27^ through institutional mailing lists at the University of Birmingham and Sandwell and West Birmingham NHS Trust. It was open from 8 May to 9 June 2022. As invitations were disseminated through open channels, the denominator and response rate were not available; this sample is also considered a convenience sample.

### Statistical analysis

FOI responses were categorised as: (1) explicit policies supporting clinical trial leave, (2) no specific policy or (3) reference to broader discretionary leave. Survey data were exported to Microsoft Excel (Microsoft Corporation, Redmond, Washington) and analysed in SPSS V.26.0 (IBM, Armonk, New York). Descriptive statistics summarised demographics and responses.

Associations between demographic variables and willingness were assessed using χ² tests. Spearman’s rank correlation examined associations between IMD decile and willingness outcomes. Multivariable logistic regression identified predictors of willingness, adjusting for demographic and employment variables. ORs with 95% CIs were reported, with p<0.05 considered significant. Analyses were exploratory due to non-probability sampling.

### Patient and public involvement

Patients and the public were not involved in the study’s design, conduct or reporting. The study focused on organisational policy and population-level attitudes rather than on a clinical intervention. Findings will be disseminated through open-access publication.

## Results

### FOI findings

**Figure 1:**
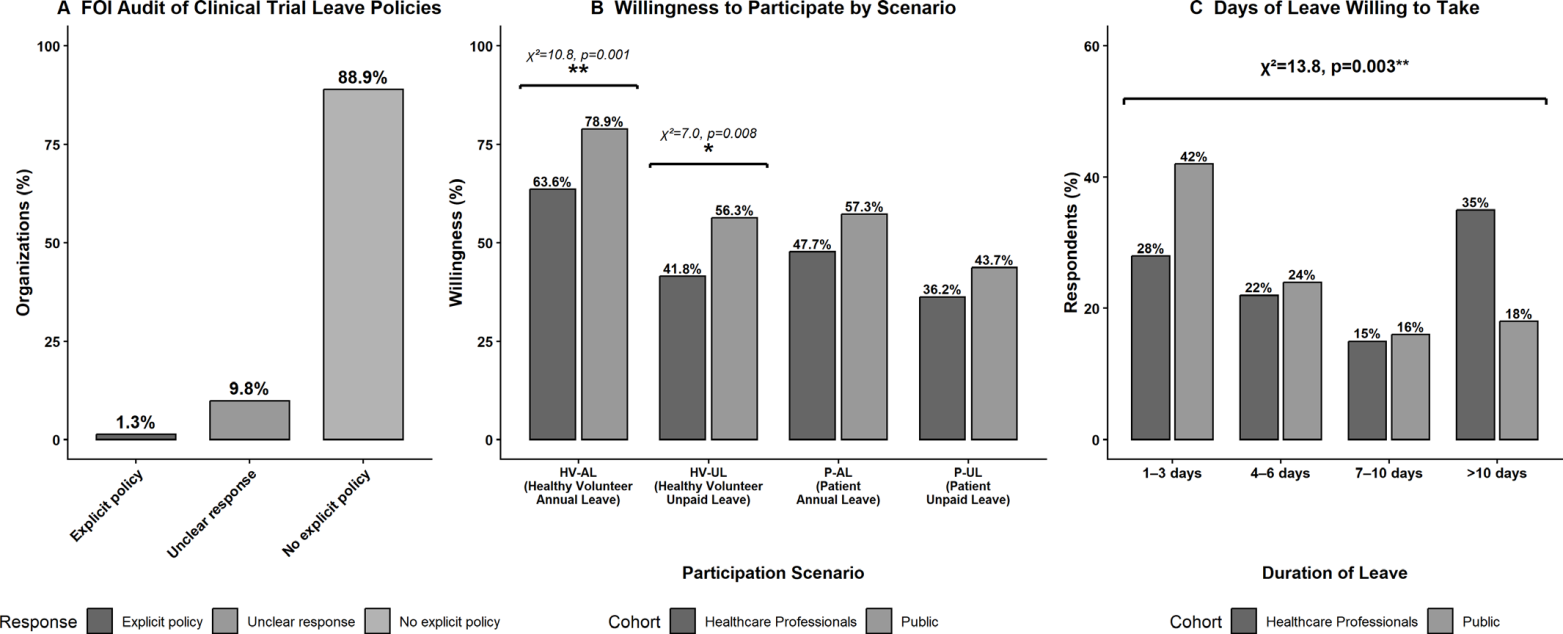
Organisational, public and healthcare professionals’ perspectives on trial participation. (**A**) Freedom of Information (FOI) audit showing the proportion of English public organisations with explicit clinical-trial leave policies. (**B**) Willingness to participate in clinical trials by scenario: healthy volunteer (HV) or patient (**P**) under annual (AL) or unpaid leave (UL). (**C**) Number of days respondents were willing to take off work for research participation. χ² and p values represent between-group comparisons. HP, healthcare professionals

FOI requests were sent to 378 organisations, and all 378 were included in the final FOI response/outcome dataset (response/outcome rate 100%). As shown in figure 1A, the majority (336 organisations; 88.9%) reported no explicit policy supporting leave for clinical trial participation. A further 37 organisations (9.8%) gave ambiguous responses, typically stating that leave would be considered on a case-by-case basis or at managerial discretion.

Only five organisations (1.3%) reported having an explicit policy, all of which were local authorities (Barnsley, Doncaster, Dudley, Cornwall and Isle of Wight Borough Councils). A review of their publicly available human resources (HR) materials indicated that none provided a dedicated or protected entitlement to participate in research. Instead, trial involvement typically fell under generic provisions, such as special leave (at managerial discretion), medical or appointment leave (for patient-participants), flexitime, AL or UL. For example, Barnsley and Dudley Councils cited ‘special leave’ for civic and personal duties, while Doncaster highlighted flexible use of AL. The Isle of Wight explicitly permitted time off for blood donation, suggesting a precedent for health-related civic activities. However, none of these policies provided protected leave comparable to that for statutory civic duties, such as jury service. No meaningful differences in response patterns were observed by organisation type; a full breakdown is provided in online supplemental appendix 1.

### Survey demographics

A total of 503 members of the public and 110 HPs completed the surveys (table 1). The public sample was broadly gender-balanced (50.9% female), whereas HPs were predominantly female (75.5%). Public respondents were distributed across age groups, with the largest proportion aged 46–55 years (23.4%), while HPs skewed younger, most commonly aged 26–35 years (31.8%). Most respondents in both groups were in full-time employment. Ethnic diversity was greater among HPs (44.5% from minority ethnic groups) than in the public sample (25.8%). Participants spanned a wide socioeconomic range, with a median IMD decile of 5 (IQR 3–8) for the public and 4 (IQR 2–7) for HPs.

**Table 1:** Demographic characteristics of public respondents and healthcare professionals

Characteristic	Combined (n=613)	Public (n=503)	HPs (n=110)	Test, p value
Gender	Female: 339 (55.3%)	Female: 256 (50.9%)	Female: 83 (75.5%)	χ² = 26.47 (df=2), p<0.001
	Male: 270 (44.0%)	Male: 245 (48.7%)	Male: 25 (22.7%)	
	Other/PNTS: 4 (0.7%)	Other/PNTS: 2 (0.4%)	Other/PNTS: 2 (1.8%)	
Age	18–25: 85 (13.9%)	18–25: 77 (15.4%)	18–25: 8 (7.3%)	χ² = 29.36 (df=5), p<0.001
	26–35: 139 (22.7%)	26–35: 104 (20.6%)	26–35: 35 (31.8%)	
	36–45: 131 (21.4%)	36–45: 108 (21.4%)	36–45: 23 (20.9%)	
	46–55: 139 (22.7%)	46–55: 118 (23.4%)	46–55: 21 (19.1%)	
	56–65: 115 (18.8%)	56–65: 98 (19.4%)	56–65: 17 (15.5%)	
	66+: 4 (0.7%)	66+: 0 (0%)	66+: 4 (3.6%)	
Work pattern	Full-time: 407 (66.5%)	Full-time: 325 (64.6%)	Full-time: 82 (74.5%)	χ² = 12.86 (df=3), p=0.005
	Part-time: 137 (22.4%)	Part-time: 114 (22.7%)	Part-time: 23 (20.9%)	
	Self-employed: 49 (8.0%)	Self-employed: 49 (9.7%)	Self-employed: 0 (0%)	
	Other: 20 (3.3%)	Other: 15 (3.0%)	Other: 5 (4.6%)	
Ethnicity	White British: 434 (70.8%)	White British: 373 (74.2%)	White British: 61 (55.5%)	χ² = 14.48 (df=1), p<0.001
	Minority: 179 (29.2%)	Minority: 130 (25.8%)	Minority: 49 (44.5%)	
Index of Multiple Deprivation Decile	Median: 5 (IQR 3–8)	Median: 5 (IQR 3–8)	Median: 4 (IQR 2–7)	U=27 892, p=0.083

Significant results are indicated at p<0.05.

HP, healthcare professionals; PNTS, prefer not to say.

### Perceptions of employer policies

Perceptions of organisational support for clinical trial participation (table 2). Only 21.4% of public respondents and 39% of HPs believed their employer had a relevant policy. In contrast, around half of the public (50.3%) and over one-third of the HPs (37.3%) believed that no such policy existed, while uncertainty was common in both groups (28.3% and 23.7%, respectively).

**Table 2:** Summary of perceived employer policies regarding leave for clinical trial participation

Question	Response	Combined (n=613)	Public (n=503)	HPs (n=110)	χ² (df), p value
Are you aware if your employer has a policy to allow paid time away for Clinical Trials appointments, if you were taking part as a healthy volunteer or as a patient?	Yes	151 (24.6%)	108 (21.4%)	43 (39.0%)	χ² = 15.26 (df=2), p<0.001
	No	294 (48.0%)	253 (50.3%)	41 (37.3%)	
	Don’t know	168 (27.4%)	142 (28.3%)	26 (23.7%)	
Do you believe your employer would allow you to receive 5–7 days of paid leave (in addition to your annual leave) per year for Clinical Trial appointments, if you were taking part as a healthy volunteer or as a patient?	Yes	175 (28.5%)	151 (30.0%)	24 (22.0%)	χ² = 38.04 (df=2), p<0.001
	No	191 (31.2%)	176 (35.0%)	15 (13.6%)	
	Don’t know	237 (38.7%)	166 (33.0%)	71 (64.4%)	

Survey results show responses from the general public and healthcare professionals (HPs). Significant results are indicated at p<0.05. For the second employer-policy question, 10 public respondents selected ‘prefer not to say’; percentages are calculated using the full cohort denominator.

When asked whether their employer would permit 5–7 days of protected leave for clinical trial participation, responses varied. Among the public, views were divided: 30.0% believed this would be permitted, 35.0% believed it would not and 33.0% were unsure. Uncertainty was particularly pronounced among HPs, with 64.4% selecting ‘don’t know’.

### Willingness to participate and acceptable leave duration

Overall, willingness to participate in clinical trials was high but varied by leave type and cohort. Public respondents consistently reported higher willingness than HPs across all scenarios. The highest willingness was observed for HV participation using AL (HV–AL), reported by 78.9% of the public and 63.6% of HPs (χ²=10.8, p=0.001). Willingness declined substantially when UL was required (HV–UL: 56.3% vs 41.8%; χ²=7.0, p=0.008). Willingness was lower overall for patient scenarios (P–AL 57.3% vs 47.7%; P–UL 43.7% vs 36.2%), although between-group differences were not statistically significant (all p>0.05) (figure 1B).

Acceptable leave duration also differed by cohort. As shown in figure 1C, public respondents were more likely than HPs to report willingness to commit longer periods of leave for clinical trial participation. More than 7 days’ leave was acceptable to 35% of public respondents compared with 18% of HPs, whereas 42% of HPs would commit no more than 3 days, compared with 28% of public respondents (χ²=13.8, p=0.003).

### Subgroup and adjusted analyses of willingness

To assess demographic influences on willingness to participate, responses from the public (n=503) and HP (n=110) cohorts were pooled (total n=613). Willingness was highest under AL scenarios (HV–AL 76.4%, n=468; P–AL 55.6%, n=341) and declined under UL conditions (HV–UL 53.3%, n=327; P–UL 42.6%, n=261).

Unadjusted analyses identified employment group as the only significant factor under the HV–UL scenario, with public respondents more willing to participate than HPs (56.3% vs 41.8%; χ²=7.0, p=0.008). No consistent associations were observed for gender, age, ethnicity or work pattern.

Socioeconomic deprivation, measured by IMD decile, was not associated with willingness in any scenario. Among public respondents, correlations between IMD and willingness were close to zero across all participation modes (all |ρ|≤0.05, p>0.25). Findings were similar among HPs and in the combined dataset, with no consistent or reproducible associations.

Multivariable logistic regression confirmed employment group as an independent predictor of willingness. Compared with public respondents, HPs had lower odds of willingness under several scenarios: HV–AL (aOR 0.52, 95% CI 0.32 to 0.85; p=0.009), P–AL (aOR 0.67, 95% CI 0.44 to 0.98; p=0.039), and P–UL (aOR 0.61, 95% CI 0.38 to 0.96; p=0.032). The HV–UL model showed the same direction of effect but was not statistically significant (p=0.082). IMD decile was not a significant predictor in any model, and no consistent effects were observed for gender, age, ethnicity or work pattern.

### Reasons for non-participation

Among respondents unwilling to use AL (table 3, section A), the most common reasons were reserving leave for family or social commitments (public 45.1%; HPs 43.8%) and holidays (41.3% and 39.1%, respectively). Many respondents also expected compensation (public 56.1%; HP 52.0%). 48.2% of HVs stated they would want to be paid for their time.

**Table 3:** Barriers to clinical trial participation

Section	Barrier/theme	Public (n=503)	HPs (n=110)
(A) Reasons for refusing annual leave (AL)	AL is for family/friends	227 (45.1%)	48 (43.8%)
	AL is for holidays	208 (41.3%)	43 (39.1%)
	Want to be paid for my time	Ps: 282 (56.1%) HVs: 243 (48.2%)	57 (52.0%)
(B) Themes from unpaid leave (UL) free-text responses	Financial concerns	HVs: 174 (34.6%) Ps: 207 (41.2%)	HVs: 60 (54.8%) Ps: 64 (58.1%)
	Work-related barriers	HVs: 207 (41.1%)	HVs: 34 (30.5%)
	Incentives (motivation, altruism, interest)	HVs: 85 (16.8%) Ps: 113 (22.4%)	HVs: 23 (21.0%) Ps: 29 (26.0%)
	Other (risk perception, lack of information)	50–75 (10–15%) across scenarios	13–20 (12–18%) across scenarios

Section A summarises structured survey responses regarding use of AL for participation. Section B presents themes derived from free-text responses describing barriers under UL scenarios.

HP, healthcare professionals; HV, healthy volunteer; Ps, patient participants.

Free-text responses from HPs frequently emphasised fairness, with several describing the use of personal leave for research as ‘inappropriate’.

For UL (table 3, section B), financial concerns dominated, particularly among public respondents, who reported an inability to afford the loss of pay (HV 34.6%; Ps 41.2%). Work-related constraints (41.1% of public HVs), flexible working and limited employer support were also common. Reimbursement and travel coverage were mentioned by 16.8% of HVs and 22.4% of Ps.

HPs also cited financial barriers, particularly for patient roles (58.1%), but qualitative responses again highlighted concerns about fairness and principle. Several HPs described annual or UL for research as ‘inappropriate’. Incentives were noted by 21% of HVs and 26% of Ps. Across both groups, additional themes included concerns about potential side effects and insufficient information about trial processes.

## Discussion

### Principal findings

This mixed-methods study highlights that although willingness to participate in clinical trials is high in principle, it is highly contingent on the type of leave available and the policy environment. This study’s most important finding is systemic: the FOI requests revealed that only 1.3% of the English public organisations contacted have explicit clinical trial leave policies, creating a mismatch between expressed willingness and the feasibility of participation, and a structural omission within UK employment frameworks. Importantly, the FOI results show that trial leave is not systematically recognised as a civic entitlement in the UK, unlike other forms of protected leave, such as jury service.

The attitudinal surveys contextualise this gap, showing that willingness to participate is high but collapses when UL is required. Public respondents were significantly more willing than HPs to participate as HV under AL, but willingness fell sharply in UL scenarios. The thematic analysis underscored this divergence: members of the public emphasised financial hardship and the need for reimbursement, whereas HPs emphasised the fairness of sacrificing scarce AL.

### Comparison with previous research

Our findings align with prior evidence that financial, time and logistical barriers are among the most commonly cited reasons for declining trial participation.^12–15 18^ Systematic reviews confirm that loss of earnings, travel expenses and leave requirements frequently deter participation across diverse populations.^17 18^ However, most previous studies have examined patient-level perceptions or study-level design features. Very few have investigated the employment policy context—a determinant we show to be highly consequential.^15 28^

The greater reluctance among HPs in this study echoes earlier qualitative work suggesting that clinicians may feel overburdened, with trial participation perceived as an additional workload or a distraction from clinical priorities.^29^ By contrast, the public framed participation in terms of financial viability, reflecting cost-of-living pressures and the opportunity costs of unpaid time.

### Civic duty and social contract framing

One finding was the spontaneous framing of clinical trial participation as a civic duty, likened to jury service. Jury service is legally protected: employees are entitled to time off and compensation, and employers cannot penalise staff for attending court.^7^ By contrast, no such entitlement exists for participation in clinical trials, despite trials being fundamental to medical progress.

Framing research participation as a civic good could reshape policy discourse. There is a growing debate across Europe about statutory volunteering leave: in Spain, employees are entitled to paid leave for civic duties and volunteering,^30^ whereas UK civil society organisations have urged the government to legislate for statutory volunteering leave.^31^ Civil service HR policies already make allowances for roles, such as magistrates or school governors.^32^ In this context, the absence of provision for clinical trial participation appears anomalous. Just as society accepts temporary disruption for jury service to sustain the justice system, so too could temporary disruption for clinical trial participation be legitimised as essential for sustaining healthcare innovation. Without such recognition, participation remains a ‘private choice’ disproportionately borne by those with financial or employment flexibility, thereby embedding inequities.

Expanding the civic duty framing, public trust and engagement in research are increasingly recognised as vital to trial success. Transparent policies and visible employer support can foster a culture in which participation is seen not only as a personal choice but also as a contribution to collective health. Embedding civic identity into trial participation may help normalise involvement and reduce stigma or hesitation.

### Potential implications for ophthalmology

The survey scenarios assessed willingness to participate in clinical trials generally rather than ophthalmology-specific studies. The ophthalmology implications discussed here should, therefore, be interpreted as a clinically relevant application of the findings to a specialty in which repeated hospital attendance, prolonged imaging, visual function testing, pupil dilation and carer involvement may increase participation burden, rather than as effects directly demonstrated by ophthalmology-specific survey data.

Uveitis (intraocular inflammation) accounts for 10%–15% of legal blindness in the developed world and provides a clinically relevant example of a condition in which employment conditions may plausibly influence the feasibility of clinical trial participation.^33^ Although uveitis may affect any age group, it peaks in the working-age population, many of whom balance employment responsibilities, variable disease activity and recurrent specialist follow-up.^34^ Trial participation typically requires repeated, lengthy visits to hospital eye units for multimodal imaging and visual function testing, which cannot be performed in community settings. These visits often last several hours and may involve pupil dilation, thereby preventing driving or returning to work on the same day. For many patients, attendance also requires a carer, introducing a second layer of potential lost income or leave.

In this context, participation can effectively become means-tested: individuals with secure employment, flexible working arrangements or paid leave may be better placed to participate, whereas those in lower paid or inflexible roles may face disproportionate exclusion. However, in the present study, IMD decile was not associated with willingness to participate. This may reflect the fact that IMD is an area-level measure and may not capture individual employment security, access to paid leave, contractual flexibility, caring responsibilities or the ability to absorb short-term income loss. Systematic reviews of clinical trials in uveitis have highlighted recruitment difficulties, retention challenges and the high logistical burden of participation.^35 36^ In addition, older adults, who may represent the epidemiologically typical population in a clinical trial, face distinct barriers, including multimorbidity, reduced mobility and reliance on working-age carers. Evidence from early-phase clinical trials shows that older adults can and do participate safely when enrolled, but are less likely to take part because of logistical, functional and support-related constraints rather than unwillingness.^37–39^ These findings reinforce that participation, among both working age and older adults, is often a function of structural feasibility rather than patient motivation. Further research examining trial discontinuation patterns stratified by age, employment status and caring roles would clarify the extent to which work-related constraints underpin these barriers. A further practical consideration in ophthalmology is that many ocular lubricants and surface agents are classified as class IIa medical devices under the *Medicines and Healthcare products Regulatory Agency’s* risk-based classification algorithm. This means that early-phase clinical evaluation requires enhanced monitoring and reporting protocols, potentially increasing the perceived burden and risk from the patient’s perspective.^40 41^ These combined factors: working-age disease, high visit burden, carer involvement and regulatory classification effects, make ophthalmology, particularly uveitis, a strong candidate for piloting protected research leave and structured reimbursement.

### Policy implications

The findings of this study indicate that barriers to clinical trial participation are not solely informational or motivational but are also shaped by workplace conditions and employment policy. Table 4 summarises four policy areas with potential to support equitable participation, including the introduction of protected research leave, standardised reimbursement practices, pilot schemes within public-sector employers and framing participation as a civic contribution. These proposals aim to reduce structural barriers while preserving ethical safeguards and proportionality. In a grant application, this could be costed using a microcosting framework combining protocol-defined visit frequency and duration with travel time, dilation-related inability to return to work, carer attendance, participant occupation, employer labour-cost assumptions and sponsor reimbursement. For example, an illustrative uveitis trial requiring 6 weekday visits, each resulting in one full working day away from work because of travel, hospital attendance, multimodal imaging, visual function testing, clinical review and pupil dilation, would equate to approximately 45 hours of work absence per participant. Applying the Office for National Statistics estimate of average labour compensation per hour worked of approximately £29 gives an estimated employer cost of £1305, rounded to £1300 per working participant.^42^ If a working-age carer is also required, the employer-level productivity cost may increase further before accounting for sponsor reimbursement, travel costs, replacement staffing, missed visits, delayed recruitment or trial extension costs. Such modelling could be built prospectively into trial budgets and used to compare the cost of protected research leave with the costs of recruitment delay, attrition and under-representation.

**Table 4:** Summary of policy recommendations to support clinical trial participation

Recommendation	Rationale	Potential stakeholders	Feasibility considerations
Introduce statutory ‘research participation leave’	Trial participation is not currently recognised as a civic duty, unlike jury service. Protected leave would reduce structural barriers.	UK Government (DHSC, BEIS), NIHR, employers, trade unions	Paid leave for public trials could be reimbursed via tax relief or direct subsidy. Unpaid guaranteed leave for industry trials avoids employer cost burden. Pilot schemes could test uptake and cost.
Standardise reimbursement and travel support	Financial barriers deter participation. Consistent reimbursement avoids inequity and undue inducement.	NIHR, HRA, trial sponsors, NHS Trusts	Align with HMRC mileage rates and NIHR/HRA guidance. Minimal cost if embedded in trial budgets. Improves transparency and trust.
Pilot leave schemes in NHS and public sector organisations	NHS is both a major employer and research host. Pilots can test feasibility and impact.	NHS England, NHS Trust HR departments, NIHR BRCs	Low-cost pilots could use existing discretionary leave frameworks. Evaluation could include recruitment rates, retention and diversity.
Link participation incentives to civic identity	Framing participation as a civic act may improve engagement and normalise leave use.	NIHR, DHSC, public health campaigns, employers	Low-cost communications strategy. Ethical framing avoids promotional recruitment. Could include symbolic recognition (eg, certificates, thank-you letters).

BEIS, Department of Business, Energy & Industrial Strategy; BRCs, Biomedical Research Centres; DHSC, Department of Health and Social Care; HMRC, HM Revenue & Customs; HR, Human Resources; HRA, Health Research Authority; NHS, National Health Service; NIHR, National Institute for Health and Care Research.

### Strengths and limitations

Strengths include the use of FOI data across a wide organisational base and triangulation with two parallel surveys (public and HPs). The mixed-methods design enabled quantitative findings to be enriched and contextualised through qualitative insights.

Limitations include that FOI requests were restricted to public-sector organisations, although private employers may also lack explicit trial-leave policies. Both surveys relied on self-reported willingness rather than observed behaviour, were subjected to sampling bias and may reflect varied interpretations of what constitutes a ‘clinical trial’. The survey findings should be interpreted cautiously. Both surveys used non-probability sampling, conventional response rates could not be calculated, and the HP sample was relatively small (n=110) and regionally recruited rather than national. The public survey did not include respondents aged >65 years, consistent with the study’s focus on working-age adults for whom employer policies are relevant, but this limits generalisability to older adults. Occupation was not captured in the public survey, so some respondents may have been HPs. The HP sample was drawn from a single regional network and may not be representative of the wider NHS workforce. Regression analyses of unpaid-leave outcomes were constrained by sparse data. Finally, the online survey format may under-represent individuals with limited digital access or digital literacy.

### Future research

Future research should assess the feasibility and impact of protected research participation leave through pilot schemes in real-world employment settings. These studies could examine effects on recruitment rates, participant diversity, retention and trial delivery costs. Clinical trials impose a high visit burden on working-age adults as well as on older patients who may depend on working-age carers to attend frequent study appointments. Work-related barriers may also disproportionately affect individuals from minority ethnic backgrounds, who are over-represented in lower paid, inflexible or precarious employment, thereby exacerbating inequities in access to research participation.^43 44^ Mapping how work constraints intersect with age, caring roles, ethnicity and socioeconomic disadvantage could help identify where protected leave or structured reimbursement would deliver the greatest benefit. Economic modelling could also quantify the cost implications for employers, trial sponsors and the health system, thereby supporting scalable policy design. The illustrative framework described above could be tested prospectively within trial feasibility studies or pilot protected-leave schemes.

## Conclusion

This study shows that willingness to participate in clinical trials is high yet fragile, shaped by leave type and the systemic policy context. Without explicit organisational or statutory protections, participation disproportionately excludes those unable to forgo income or AL. Framing trial participation as a civic duty and introducing modest protected leave entitlements could improve recruitment, equity and representativeness, with particular benefit for ophthalmology, where trial burdens are high and patient populations are vulnerable.

## Supplementary material

10.1136/bmjophth-2026-002903 online supplemental file 1

10.1136/bmjophth-2026-002903 online supplemental file 2

10.1136/bmjophth-2026-002903 online supplemental file 3

10.1136/bmjophth-2026-002903 online supplemental file 4

## Data Availability

Data are available upon reasonable request.
