# Supplementary material for: Bridging policy gaps in clinical trial volunteering: public and healthcare professional perspectives in England
Source: BMJ Open Ophthalmol. 2026 Jul 28;11(3):e002903. doi: 10.1136/bmjophth-2026-002903 (PMC13422919; doi:10.1136/bmjophth-2026-002903)
Supplement: online supplemental file 2 [file bmjophth-11-3-s002.pdf]

Healthcare Professionals

Public Attitudes to Clinical Trial Involvement

Before completing this survey, please view this 2 minute video;

Public attitudes to Clinical Trial involvement - YouTube

This survey should take no longer than 5 minutes to complete.

Thank you for taking the time to complete this survey.

Clinical Trials are a way for researchers to find out if a new treatment, like a new drug or medical device (for example, a pacemaker) is safe and effective. Clinical Trials may involve making multiple visits to a hospital/GP Surgery. For example, in some Clinical Trials you may have to be seen every day, a number of times a week, once a week/month for a number of months and then possibly every 3-6 months depending on the duration of the trial.

1. What is your Current Working Pattern?
   - ◯ Full Time
   - ◯ Part Time
   - ◯ Self Employed
   - ◯ Flexible Working
   - ◯ Shift Working
   - ◯ Zero Hour Contract
   - ◯ Not Currently Employed
   - ◯ Prefer Not to Say

2. Age
   - ◯ 18-25
   - ◯ 26 -35
   - ◯ 36 - 45
   - ◯ 46- 55
   - ◯ 56 - 65
   - ◯ 66-75
   - ◯ 76 +
   - ◯ Prefer not to say

3. Gender
   - ◯ Male
   - ◯ Female
   - ◯ Prefer not to say

4. Ethnicity

- ◯ Asian or Asian British - Bangladeshi
- ◯ Asian or Asian British - Chinese
- ◯ Asian or Asian British - Indian
- ◯ Asian or Asian British - Pakistani
- ◯ Asian or Asian British - Any other Asian background
- ◯ Black or Black British - African
- ◯ Black or Black British - Caribbean
- ◯ Black or Black British - Any other Black, African or Caribbean background
- ◯ Mixed or Multiple ethnic groups - White and Black African
- ◯ Mixed or Multiple ethnic groups - White and Asian
- ◯ Mixed or Multiple ethnic groups - White and Black Caribbean
- ◯ Mixed or Multiple ethnic groups - Any other Mixed or Multiple ethnic background
- ◯ Other ethnic group - Arab
- ◯ Other ethnic group - Any other ethnic group
- ◯ White - English, Welsh, Scottish, Northern Irish or British
- ◯ White - Irish
- ◯ White - Gypsy or Irish Traveller
- ◯ White - Any other White background
- ◯ Other
- ◯ Prefer not to say

5. What is your full Postcode ______________________

PART 1:

In this section imagine you are a HEALTHY VOLUNTEER (someone with no known health problems) that is eligible to take part in a clinical trial. As a healthy volunteer you will be PAID for your time. This can vary from hundreds to thousands of pounds depending on what is involved and what is expected of you.

6. If you were eligible to take part in a Clinical Trial as a HEALTHY VOLUNTEER (you will get paid for your participation). Would you be willing to take ANNUAL LEAVE from work to participate?

◯ Yes
◯ No

6a. How many days of ANNUAL LEAVE would you take as a HEALTHY VOLUNTEER in a Clinical Trial in a year?

◯ 1-3
◯ 4-6
◯ 7-10
◯ 10+

6b. Reason(s) why? Please tick all that apply.

☐ Annual Leave is for holidays
☐ Annual leave is for time to spend with family/friends
☐ Other
☐ Prefer not to say

6c. Other

____

7. If you were eligible to take part in a Clinical Trial as a HEALTHY VOLUNTEER (You will get paid for your participation). Would you be willing to take UNPAID LEAVE from work to participate?

◯ Yes
◯ No

7a. How many days of UNPAID LEAVE would you take as a HEALTHY VOLUNTEER in a Clinical Trial in a year?

◯ 1-3
◯ 4-6
◯ 7-10
◯ 10+

7b. Reason(s) why?

____

PART 2

In this section imagine you are a PATIENT suffering with a medical condition who has been asked to take part in a Clinical Trial. As a patient you will NOT be paid for your time and will only receive travel expenses.

8. If you were eligible to take part in a Clinical Trial as a PATIENT (no payment provided, only travel expenses). Would you be willing to take ANNUAL LEAVE from work to participate?

◯ Yes
◯ No

8a. How many days of ANNUAL LEAVE Would you take as a PATIENT in a Clinical Trial in a year?

◯ 1-3
◯ 4-6
◯ 7-10
◯ 10+

8b. Reason(s) why? Please tick all that apply.

☐ I want to be paid for my time
☐ Annual leave is for going away
☐ Annual Leave is for time with Family/Friends
☐ Other
☐ Prefer not to say

8c. Other

____

9. If you were eligible to take part in a Clinical Trial as a PATIENT (no payment provided, only travel expenses). Would you be willing to take UNPAID leave from work to participate?

◯ Yes
◯ No

9a. How many days of UNPAID LEAVE would you take as a PATIENT in a Clinical Trial in a year?

◯ 1-3
◯ 4-6
◯ 7-10
◯ 10+

9b. Reason(s) why?

____

PART 3

In this section we want to find out if you know of any policies your employer may have in place for you to take part in Clinical Trials and if you think your employer would allow you to take paid leave (in addition to your annual leave) to do so.

10. Are you aware if your employer has a policy to allow paid time away for Clinical Trials appointments, if you were taking part as a HEALTHY VOLUNTEER or as a PATIENT?
    - ◯ Yes
    - ◯ No
    - ◯ Don't know

11. Do you believe your employer would allow you to receive 5-7 days of paid leave (in addition to your annual leave) per year for Clinical Trial appointments, if you were taking part as a HEALTHY VOLUNTEER or as a PATIENT?
    - ◯ Yes
    - ◯ No
    - ◯ Don't know
    - ◯ Prefer not to say
