# Supplementary material for: Bridging policy gaps in clinical trial volunteering: public and healthcare professional perspectives in England
Source: BMJ Open Ophthalmol. 2026 Jul 28;11(3):e002903. doi: 10.1136/bmjophth-2026-002903 (PMC13422919; doi:10.1136/bmjophth-2026-002903)
Supplement: online supplemental file 3 [file bmjophth-11-3-s003.docx]

Clinical Trial Involvement Survey – Public

Before completing this survey, please view this 2 minute video;

Public attitudes to Clinical Trial involvement - YouTube

This survey should take no longer than 5 minutes to complete.

Thank you for taking the time to complete this survey.

1. What is your Current Working Pattern?

- Full Time

- Part Time

- Self Employed

- Flexible Working

- Shift Working

- Zero Hour Contract

- Not Currently Employed

- Prefer Not to Say

2. Age

- 18–25

- 26–35

- 36–45

- 46–55

- 56–65

- Prefer not to say

3. Gender

- Male

- Female

- Prefer not to say

4. Ethnicity

Asian or Asian British – Bangladeshi

Asian or Asian British – Chinese

Asian or Asian British – Indian

Asian or Asian British – Pakistani

Asian or Asian British – Any other Asian background

Black or Black British – African

Black or Black British – Caribbean

Black or Black British – Any other Black, African or Caribbean background

Mixed or Multiple ethnic groups – White and Black African

Mixed or Multiple ethnic groups – White and Asian

Mixed or Multiple ethnic groups – White and Black Caribbean

Mixed or Multiple ethnic groups – Any other Mixed or Multiple ethnic background

Other ethnic group – Arab

Other ethnic group – Any other ethnic group

White – English, Welsh, Scottish, Northern Irish or British

White – Irish

White – Gypsy or Irish Traveller

White – Any other White background

Other

Prefer not to say

5. Occupational group

- Semi or unskilled manual work

- Skilled manual worker

- Supervisory or clerical / junior managerial / professional / administrative

- Intermediate managerial / professional / administrative

- Higher managerial / professional / administrative

- Student

- Casual worker – not in permanent employment

- Homemaker

- Retired and living on state pension

- Unemployed or not working due to long‑term sickness

- Carer of other household member

- Other

- None of these

6. What is your full postcode?

____________________________________

PART 1 – Healthy Volunteer Scenario

7. Willing to take ANNUAL LEAVE to participate?

- Yes

- No

7a. How many days of annual leave?

- 1–3

- 4–6

- 7–10

- 10+

7b. Reasons (tick all that apply):

- Annual Leave is for holidays

- Annual Leave is for time to spend with family/friends

- Other

- Prefer not to say

7c. Other (free text):

____________________________________

8. Willing to take UNPAID LEAVE to participate?

- Yes

- No

8a. How many days of unpaid leave?

- 1–3

- 4–6

- 7–10

- 10+

8b. Reason(s) why?

____________________________________

PART 2 – Patient Scenario

9. Willing to take ANNUAL LEAVE to participate?

- Yes

- No

9a. How many days?

- 1–3

- 4–6

- 7–10

- 10+

9b. Reasons (tick all that apply):

- I want to be paid for my time

- Annual leave is for going away

- Annual leave is for time with family/friends

- Other

- Prefer not to say

9c. Other:

____________________________________

10. Willing to take UNPAID LEAVE to participate?

- Yes

- No

10a. How many days?

- 1–3

- 4–6

- 7–10

- 10+

10b. Reason(s) why?

____________________________________

PART 3 – Employer Policies

11. Aware of employer policy?

- Yes

- No

- Don’t know

12. Would employer allow 5–7 days paid leave for clinical trial appointments?

- Yes

- No

- Don’t know

- Prefer not to say
